# Impact of human disturbance on bee pollinator communities in savanna and agricultural sites in Burkina Faso, West Africa

**DOI:** 10.1002/ece3.4197

**Published:** 2018-06-17

**Authors:** Katharina Stein, Kathrin Stenchly, Drissa Coulibaly, Alain Pauly, Kangbeni Dimobe, Ingolf Steffan‐Dewenter, Souleymane Konaté, Dethardt Goetze, Stefan Porembski, K. Eduard Linsenmair

**Affiliations:** ^1^ Department of Animal Ecology and Tropical Biology Biocenter University of Wuerzburg Wuerzburg Germany; ^2^ Department of Botany and Botanical Garden Institute of Biological Sciences University of Rostock Rostock Germany; ^3^ Faculty of Organic Agricultural Sciences Universität Kassel Kassel Germany; ^4^ Unité de Formation et de Recherche des Sciences de la Nature Unité de Recherche en Ecologie et Biodiversité Université Nangui Abrogoua Abidjan Côte d'Ivoire; ^5^ Department of Entomology Royal Belgian Institute of Natural Sciences Brussels Belgium; ^6^ Laboratoire de Biologie et Ecologie Végétales UFR/SVT Université Ouaga1 Pr Joseph Ki‐Zerbo Ouagadougou Burkina Faso

**Keywords:** bee communities, cotton, sesame, species spillover, sub‐Saharan Africa

## Abstract

All over the world, pollinators are threatened by land‐use change involving degradation of seminatural habitats or conversion into agricultural land. Such disturbance often leads to lowered pollinator abundance and/or diversity, which might reduce crop yield in adjacent agricultural areas. For West Africa, changes in bee communities across disturbance gradients from savanna to agricultural land are mainly unknown. In this study, we monitored for the impact of human disturbance on bee communities in savanna and crop fields. We chose three savanna areas of varying disturbance intensity (low, medium, and high) in the South Sudanian zone of Burkina Faso, based on land‐use/land cover data via Landsat images, and selected nearby cotton and sesame fields. During 21 months covering two rainy and two dry seasons in 2014 and 2015, we captured bees using pan traps. Spatial and temporal patterns of bee species abundance, richness, evenness and community structure were assessed. In total, 35,469 bee specimens were caught on 12 savanna sites and 22 fields, comprising 97 species of 32 genera. Bee abundance was highest at intermediate disturbance in the rainy season. Species richness and evenness did not differ significantly. Bee communities at medium and highly disturbed savanna sites comprised only subsets of those at low disturbed sites. An across‐habitat spillover of bees (mostly abundant social bee species) from savanna into crop fields was observed during the rainy season when crops are mass‐flowering, whereas most savanna plants are not in bloom. Despite disturbance intensification, our findings suggest that wild bee communities can persist in anthropogenic landscapes and that some species even benefitted disproportionally. West African areas of crop production such as for cotton and sesame may serve as important food resources for bee species in times when resources in the savanna are scarce and receive at the same time considerable pollination service.

## 1 INTRODUCTION

During the last century, conversion of natural habitats and land‐use intensification at habitat and landscape scale have been the major drivers of global environmental change in terrestrial ecosystems (Sala et al., 2000). Such changes have led to landscape mosaics of both human‐managed and natural areas. When studying the impact of land‐use change and disturbance, it is of particular importance to understand whether organisms that perform important ecosystem services persist in human‐dominated ecosystems. Pollinators are one such group: Most of the world's flowering plants require animal pollinators (Ashman et al., 2004), and plant populations in human‐dominated ecosystems will only maintain genetic diversity if pollinators are present and can move freely through anthropogenic habitats (Keller & Waller, 2002). Bees are key providers of pollination services, which are vital for crop production and food security and the persistence of many wild plants (Klein et al., 2007; Ollerton, Winfree, & Tarrant, 2011). However, many bee species are threatened by land‐use intensification and human disturbance of natural habitats (Ollerton, Erenler, Edwards, & Crockett, 2014; Potts et al., 2010). Land‐use change, such as large‐scale conversion of seminatural habitats to human‐dominated landscapes, can greatly impact bee communities through reduced floral resources (Forrest, Thorp, Kremen, & Williams, 2015) and nesting sites (Shuler, Roulston, & Farris, 2005). Many pollinators visit crop habitats for foraging, but might need to return to natural habitats to complete their reproductive cycle because of the frequent disturbance regime in agricultural fields (Greenleaf, Williams, Winfree, & Kremen, 2007; Holzschuh, Steffan‐Dewenter, Kleijn, & Tscharntke, 2007). This underlines the importance of natural and seminatural habitats which can provide spillover (i.e., movement of organisms and their function between natural habitats and agricultural sites) of pollinators and their pollination services to nearby cropland and vice versa (Blitzer et al., 2012).

Although agriculture may potentially harm wild pollinator populations, bee responses to agriculture are not uniformly negative (Williams et al., 2010; Winfree, Aguilar, Vázquez, LeBuhn, & Aizen, 2009). A number of studies have shown that pollinators use, and maybe even rely on, resources from crop fields and then return to natural habitats. Hence, agricultural habitats may serve as supplementary resources promoting bee populations and hence their pollination service to wild plants and crops (Kremen et al., 2007; Lander, Bebber, & Choy, 2011).

Pollinator shortage can lead to reduced crop quality and yield, with potentially large economic impact (Kevan & Phillips, 2001). Therefore, much research has been carried out on responses of bee communities to human impacts such as land‐use change and intensification. Current data suggest an overall pattern of decline in insect diversity and abundance (Hallmann et al., 2017). This decline is likely to increase the risk of future pollination deficits in areas of high and increasing pollination demands (Aizen & Harder, 2009; Lautenbach, Seppelt, Liebscher, & Dormann, 2012). Areas where food production most highly depends on animal pollination are also those for which the fewest data are available (Archer, Pirk, Carvalheiro, & Nicolson, 2014; Gallai, Salles, Settele, & Vaissière, 2009), due to a lack of infrastructure and funding in many areas of the world, particularly in developing countries. These same areas are often poorly buffered against disruption of ecosystem service provision from whatever cause, meaning that effects of any ecological incidents on human well‐being could be more severe here than elsewhere (De Palma et al., 2016). The fact that almost half the studies on pollinator decline comes from only five countries (Australia, Brazil, Germany, Spain and USA), with only 4% of the data from the African continent (Archer et al., 2014; Winfree, Bartomeus, & Cariveau, 2011), highlights the bias in information and the lack of data from some regions. Although movements of pollinators from natural to managed agricultural landscapes have been documented across a wide range of both tropical and temperate habitats and managed landscapes (Garibaldi et al., 2011; Klein et al., 2007), most of the studies were carried out in Europe and North America. Examples from tropical regions are less available and include rainforest habitats providing resources for pollinating bees for coffee agroecosystems in Indonesia (Klein, Steffan‐Dewenter, & Tscharntke, 2003a), Costa Rica (Ricketts, 2004), Brazil (De Marco & Coelho, 2004), and Tanzania (Classen et al., 2014).

Furthermore, existing studies overrepresent bumblebees (which do not occur in most of Africa), and model results may not be generalized to other regions and taxa (De Palma et al., 2016).

Around 80% of the population in Burkina Faso relies on subsistence farming, as it is true for West Africa in general (GIZ, 2016). Burkina Faso's economic development largely depends on agriculture, with cotton (*Gossypium hirsutum* L.) as main export product (Thiombiano & Kampmann, 2010) and with sesame (*Sesamum indicum* L.) being on rank 3 among the top ten commodities export quantities of the country (FAO, 2013). Following FAO, the insect pollination economic value for West Africa is assumed to amount to 5.6 × 10^9^ USD which is highest on the entire African continent along with one of the highest vulnerability rates (Gallai et al., 2009; Potts et al., 2016).

Despite the importance of bees as pollinators and concerns about pollinator conservation, the effect of anthropogenic activities, which may be detrimental to some bee species and beneficial to others, is unknown for West African savanna ecosystems (IPBES, 2016).

Hence, the objectives of our study were (a) to assess bee species communities of savanna habitats in Burkina Faso that are characterized by a gradient in habitat disturbance, (b) to investigate the spatial relationship between bee communities of savannas and adjacent cotton and sesame fields, and (c) to analyze the seasonal movement of bees from savanna into the crop fields.

## 2 METHODS

### 2.1 Study system

**Figure 1:**
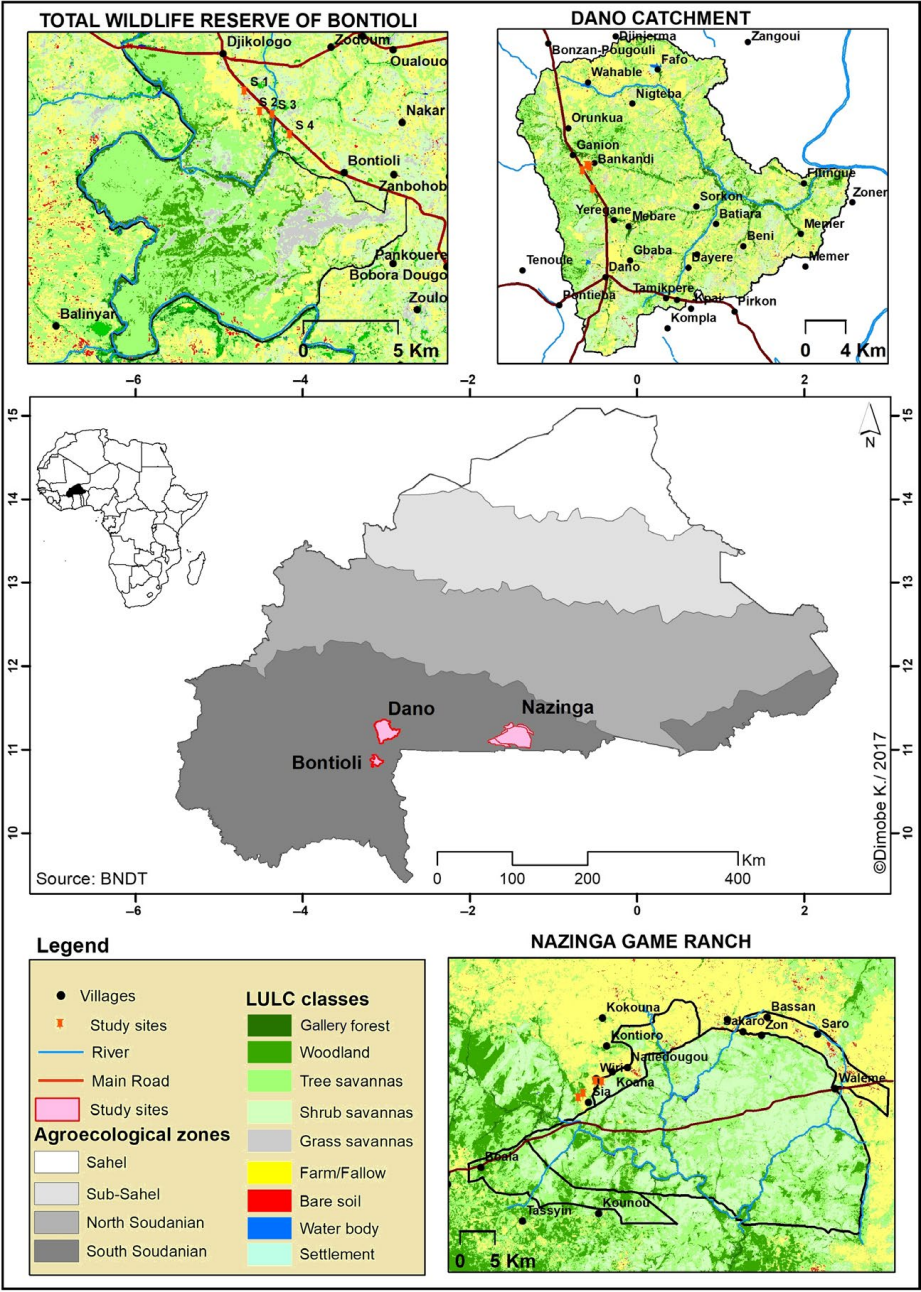
Map with land‐use and land cover data of the three study areas Nazinga (low disturbance intensity; DI), Bontioli (medium DI), and Dano (high DI) in 2014 and their location within Burkina Faso

Our study was carried out in the South Sudanian zone in Burkina Faso, sub‐Saharan West Africa (Figure 1). There are two pronounced seasons per year: a rainy season from June to October and a dry season from November to May, whereas October is a transition month between the seasons (Grote et al., 2009). Mean annual rainfall averages 800–1,000 mm (Hema, Barnes, & Guenda, 2011). Mean annual temperature is 27–28°C (MSP, 2010).

The dominant vegetation of this zone, which covers a large band in West Africa, originally consisted of woodland and savanna with a dense cover of tall grasses and varying densities of trees and shrubs (White, 1983). The latter form an open canopy and are mainly pollinated by bees. In most places where cultivation was possible, the original vegetation has been profoundly modified and replaced by mosaics of fields and fallows. The soil types in the study area are Luvisols (following the FAO soil classification) with higher clay content in the subsoil than in the topsoil. The iron enriched B horizon adds the characteristic red color. The acidic soil often is characterized by aluminum toxicity and is easily eroded. Three study areas were selected: Nazinga (11°06′34.998″ N, 001°29′07.181″ W), Bontioli (10°48′26.393″ N, 003°04′39.564″ W) and Dano (11°08′56.566″ N, 003°03′36.446″ W), at elevations between 271 and 448 m a.s.l. All study areas are located in the South of Burkina Faso and are characterized by a mosaic of agricultural land, villages and fragments of near‐natural savanna (Figure 1). They were chosen according to their differences in disturbance intensity (DI), that is the percentage of forest cover (including grass, shrub and tree savannas) and cropland cover (farms and fallows) at a landscape scale. The study areas were classified according to a 3‐point scale with low, medium and high DI based on land‐use/land cover data via multitemporal Landsat images (for methods details see Dimobe et al., 2015, 2017).

The Nazinga Game Ranch (in the following referred to as “Nazinga area”) is a protected area, classified as “Wildlife Reserve” according to Burkina Faso's legislation. It spreads over an area of 97,536 ha (Hema et al., 2011) and is characterized by tree species typical of pristine savanna forests, such as *Terminalia macroptera* Guill. & Perr., *Detarium microcarpum* Guill. & Perr. and *Prosopis africana* (Guill. & Perr.) Taub. Human disturbance is low except for regular, managed fires at the beginning of the dry season and only small settlements with agricultural fields at the margin of the reserve. The forest cover amounts to 88.2%, cropland to 0.8% (Dimobe et al., 2017). We considered disturbance in this area as “low.”

The Bontioli Nature Reserve (in the following referred to as “Bontioli area”) is also a protected area, but categorized as a “Nature Reserve” according to Burkina Faso's legislation (Tia, 2007). The Bontioli savanna spreads over an area of 25,000 ha and is characterized by dominance of the trees *Terminalia laxiflora* Engl. & Diels and *Vitellaria paradoxa* C.F. Gaertn. The DI of this area was considered as “medium” due to human activities such as agriculture, grazing, fire, uncontrolled logging and timber extraction that were registered even inside the reserve. The reserve is surrounded by plenty of villages and a wide agricultural landscape. Forest cover amounts to 77.85%, cropland to 12.59% (Dimobe et al., 2015).

The study area of Dano (in the following referred to as “Dano area”) comprises a small city of about 50,000 inhabitants with a fast growing community where mainly farmers expand their settlements more and more into the surrounding savanna. Hence, only a few, very small “near‐natural” savanna habitats have remained and only economically relevant tree species such as karité (*Vitellaria paradoxa*) and neré (*Parkia biglobosa* [Jacq.] R.Br. ex G.Don) have been left, forming a so‐called parkland landscape. Anthropogenic disturbance at the savanna sites of Dano was more intensive than at Bontioli, forming an agricultural landscape with degraded soils and intense grazing, fire and logging. Forest cover amounts to 52.9%, cropland to 37.2% (K. Dimobe, unpublished data). We therefore considered the DI in the area of Dano as “high.”

### 2.2 Data collection

In each of the three study areas (Nazinga, Bontioli and Dano) we randomly selected four savanna sites and four nearby fields of conventional upland cotton (*G. hirsutum*) and four fields of sesame (*S. indicum*), each approximately 1 ha of size. Savanna sites were not selected after certain criteria such as particular tree species occurrence or tree density. The only criterion was a minimum size of 1 ha of savanna vegetation. At each savanna site and field, four sampling plots within a grid of 60 m × 90 m (almost covering the entire size of a savanna site or field) were set up. In each plot six pan traps with a distance of 15 m between each other were installed (24 traps per site).The sampling plots were designed to ensure both identical distances between pan traps and a minimum distance of 10 m of a pan trap to the savanna or field edge, respectively.

In the highly disturbed study area of Dano only three cotton and three sesame fields could be chosen for data collection. A collaborating farmer abandoned his fields shortly after sowing. As only few scattered crop plants grew and flowered at all, these fields were not comparable with the other fields and had to be excluded from bee data collection. Thus, the total sample size for all three areas was 34 sites (12 savanna sites, 11 cotton fields and 11 sesame fields) with 136 plots.

All cotton and sesame fields had a maximum distance of 1 km to the next near‐natural savanna fragment.

Both crop types were chosen as they are the main cash crops in Burkina Faso. Cotton and sesame plants are known to be able to self‐pollinate, but in both crops outcross pollination by bees significantly enhanced yield and quality in the same study area (Stein et al., 2017). At all sites farmers were requested to continue their usual farming practice during the study period: Fertilizers were applied at the beginning of the sowing season, insecticides and fungicides were irregularly applied depending on the infestation rate and financial resources of the farmers. Weeds were removed manually.

Bee data were continuously collected from savannas over 21 months from January 2014 to September 2015, covering two dry and two rainy seasons. Bees were sampled once a month at the savanna sites and every 2 weeks at the fields, taking into account the relatively short flowering period of the crops during the rainy season. Flowering starts in late June and lasts until end of September. The flowering peak with mass‐flowering is from mid of July until end of August. Bee species that visited the cotton fields were sampled during the rainy season of 2014 and 2015; bee species that visited the sesame fields were sampled only in the rainy season of 2015 due to problems in infrastructure. Pan traps were used to sample particularly honey bees and wild bees and were placed in a height of 1 m above the ground. We installed 288 pan traps within 48 plots located each in Nazinga and Bontioli and 240 pan traps within 40 plots in Dano (in total 816 pan traps).

Each pan trap consisted of one UV‐bright yellow, white and blue 500 ml plastic bowl that was filled with salt (NaCl) saturated water and a small drop of detergent. The traps were left activated for 72 hr during each sampling turn. Specimens of bees were collected, stored in ethyl alcohol, and thereafter pinned and identified to genus or species if possible. The reference collections of the Royal Belgian Institute of Natural Sciences, Brussels, Belgium, were used to identify the species (voucher specimens of bees collected in this study are also held there).

### 2.3 Data analysis

Bee communities were analyzed regarding their abundance, estimated species richness using bootstrap estimator (Smith & van Belle, 1984) and Pielou's species evenness. Here, data were pooled per savanna and field, respectively, and data gathered from the savanna sites were additionally pooled for rainy season (June–September) and dry season (October–May). As bee data of both years did not differ significantly, both years were analyzed jointly. Savannas of the same DI) were located in close proximity wherefore omparisons between savanna types considered a potential effect of spatial autocorrelation. For this reason, we first used Moran's Test with a proximity matrix calculated from longitude and latitude values to detect if data were spatially autocorrelated. With exception of species evenness of bee communities sampled in cotton fields (Moran's test, *I *=* *0.38, *p *=* *0.026) and bee abundance within savannas during the rainy season (*I *=* *0.44, *p *=* *0.009), species data were not affected by spatial autocorrelation (see Supporting Information Table S1). For both datasets mentioned, the effect of spatial autocorrelation was removed from linear regression models using generalized least squares (GLS) while fitting the model with Gaussian autocorrelation structure. The Gaussian structure was tested to fit best our model (lowest delta Akaike Information Criterion ‐AIC).

Simple linear regression models were used to analyze to which degree bee abundance, richness and evenness of savannas were affected by DI. GLS were further used to analyze how bee communities of savannas were related to bee abundance, richness and evenness within cotton and sesame fields. We here only used bee community data that were gathered during the rainy season in 2014 within savannas and their adjacent cotton and sesame fields.

To avoid overestimation of the effect of savanna disturbance on total bee species abundance, the two most abundant bee species, namely the stingless bee *Hypotrigona gribodoi* Magretti and the western honey bee *Apis mellifera* L., were excluded from the model. A model comparison of the reduced and the full model (including both species), however, revealed no differences in our main findings. Abundance data were log‐transformed to ensure normal distribution of residuals.

Nonmetric multidimensional scaling (NMDS) was used to analyze (a) differences between bee community composition of different study areas reflecting DI and (b) differences between bee species composition in cotton and sesame fields and their respective adjacent savanna site in the year 2015. The NMDS technique is an indirect gradient analysis approach and was based on the Bray–Curtis dissimilarity matrix of bee species abundance (standardized through a Wisconsin transformation; Oksanen, 2014). For NMDS calculation all species were included.

Shannon entropy was used to assess diversity components of bee species communities found in savannas and adjacent fields together under different disturbance intensities. For each area (low, medium, high DI) we calculated *alpha* diversity (*α*_1_; mean diversity of sites) as well as first (*β*_1_; mean turnover among sites of the same habitat type) and second level of *beta* diversity (*β*_2_, mean turnover among sites of all habitats). The sum of all three components represents the regional gamma diversity (*γ*).

To investigate the seasonal movement of bee species from savanna into the crop fields (across‐habitat spillover), only bee species (and the number of individuals) that were found within both habitats within one study area (savanna *and* cotton fields; savanna *and* sesame fields) were included. All other bee species that were found exclusively in savannas (39 species) or fields (22 species), that is that did not seem to move from savanna into crop fields and vice versa, were excluded from the analysis. Abundance data of the species were pooled per site and month and standardized (*z*‐score) by subtracting the mean abundance of the joint species (savanna‐cotton, savanna‐sesame) from the individual species abundance score divided by the standard deviation of the joint species.

Statistical analyses and figure production were conducted in R version 3.2.3 (R Core Team, 2015) with additional functions provided by the R packages vegan (Oksanen et al., 2013), lme4 (Bates, Maechler, Bolker, & Walker, 2015), lmerTest (Kuznetsova, Brockhoff, & Christensen, 2016), multcomp (Hothorn, Bretz, & Westfall, 2008), plotrix (Lemon, 2006) and Mass (Venables & Ripley, 2002). Graphs were further created using SigmaStat 3.0.1, SPSS Inc. (2003).

## 3 RESULTS

A total of 35,469 bee specimens were caught during the 21 month sampling period. 28,505 specimens were recorded from savanna sites (21 months of sampling in 2014 and 2015), 5,716 from cotton fields (4 months of sampling each in 2014 and 2015) and 1,248 from sesame fields (4 months of sampling in 2015). The determined specimens found in the savannas and fields of both crop types revealed a total bee richness of 97 species assigned to 32 genera of the four families Apidae, Colletidae, Halictidae, and Megachilidae.

The dominant stingless bee species *H. gribodoi* Magretti (*n* = 25,831) was caught mainly in the medium (Bontioli area) and highly (Dano area) disturbed savannas, whereas most individuals of the subdominant species *A. mellifera* Linnaeus (*n* = 2,074) and *Seladonia lucidipennis* Smith (*n* = 1,650) were caught primarily in the savannas of Bontioli. Further common bee species were *Pseudapis interstitinervis* Strand, *Seladonia jucunda* Smith, *Tetralonia fraterna* Friese and *Meliponula togoensis* Stadelmann, of which *P. interstitinervis* was caught exclusively in savannas and not on cotton or sesame fields (see Supporting Information Table S2); contrarily, *Liotrigona* sp. 2 was solely caught in cotton fields (see Supporting Information Table S3).

**Figure 2:**
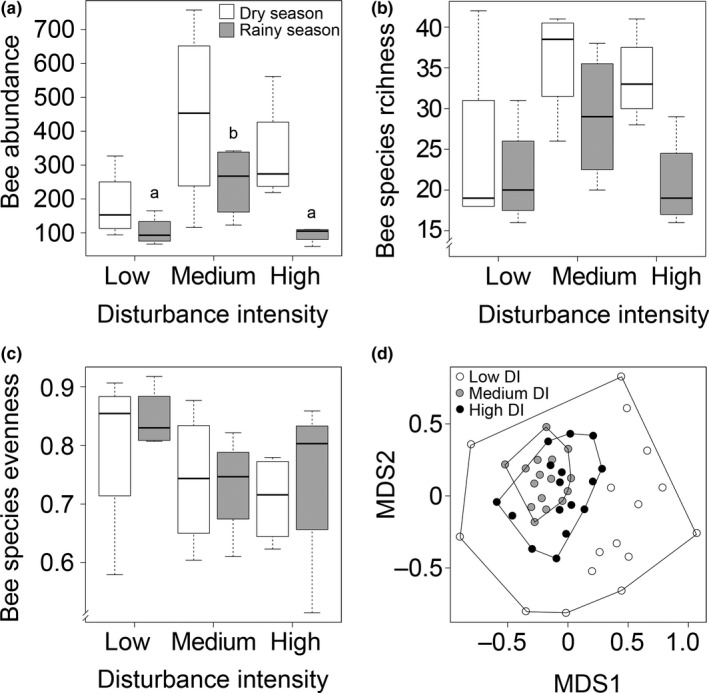
Mean total abundance (a), estimated species richness (bootstrap estimator) (b), and Pielou's species evenness (c) of bee communities caught with pan traps in savannas with low (Nazinga, *n* = 4), medium (Bontioli, *n* = 4), and high (Dano, *n* = 4) disturbance intensity (DI) in Burkina Faso during the dry and rainy season of 2014 and 2015. Different letters indicate significant differences between groups with *p *≤* *0.05. Ordination of species composition (NMDS; d) is based on a sample size of 16 savannas, cotton and sesame fields each. NMDS stress value of 0.12 and goodness of the fit of *R* ^2^ = 0.99 (nonmetric)

**Figure 3:**
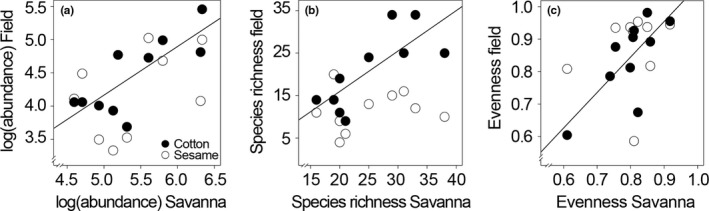
Relationship between abundance (log‐transformed), species richness (bootstrap estimator), and Pielou's species evenness of bee communities sampled in ten savannas during the rainy season 2014 and adjacent cotton fields (black dots, straight line) and sesame fields (white dots) in Burkina Faso. Regression line indicates significant relationship between parameters with *p *≤* *0.05 calculated using GLS with Gaussian correlation where necessary (Supporting Information Table S1)

The comparison of seasons revealed a significantly lower bee abundance within all savannas during the rainy season compared to the dry season (Figure 2a, GLS, *t *=* *−3.38, *p *=* *0.003). Highest abundance of bee species was recorded within medium disturbed savannas (Bontioli area), but with significant differences only for the rainy season (GLS, *t *=* *3.04, *p *=* *0.0141). Bee species richness did not differ significantly among savannas and seasons (Figure 2b). Also bee species evenness was not significantly affected by DI (Figure 2c). NMDS ordination of bee species communities revealed a high compositional heterogeneity in low disturbed savannas (Nazinga area; Figure 2d; stress: 0.12, nonmetric fit: *R* ^2^ = 0.99). In contrast, bee communities of medium (Bontioli area) and highly (Dano area) disturbed savannas were less heterogeneous and showed a high similarity to each other. A significantly positive relationship between bee abundance, species richness and evenness of savannas and their adjacent crop fields was only found for cotton fields but not for sesame (Figure 3a–c).

**Figure 4:**
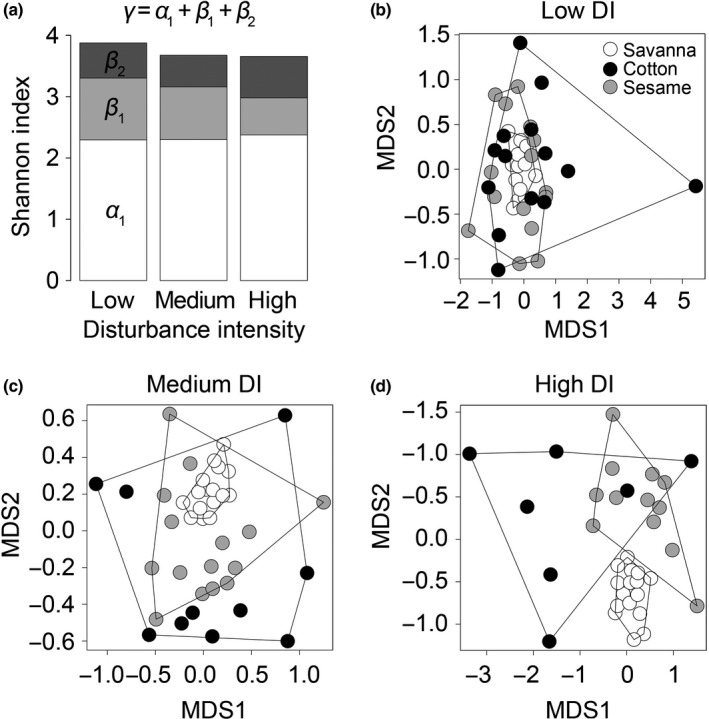
Diversity components of bee species communities under different disturbance intensities (a) and result of ordination (NMDS) of bee communities sampled in savannas (white dots; stress: 0.12, nonmetric fit, *R* ^2^ = 0.99) and adjacent cotton (dark gray dots; stress: 0.19, nonmetric fit, *R* ^2^ = 0.96) and sesame fields (light gray dots; stress: 0.16, nonmetric fit, *R* ^2^ = 0.97) under low (b), medium (c), and high (d) disturbance intensity in 2014 and 2015 in Burkina Faso

The analysis of different diversity levels (Shannon Index) of the bee species communities revealed no significant differences between low, medium and highly disturbed areas (Figure 4a). Mean alpha diversity (*α*_1_) remained unchanged with increasing DI. However, a decrease in spatial heterogeneity of bee communities of the same habitat type (*β*_1_) toward more disturbed areas was noticeable whereas spatial heterogeneity of bee communities between different habitat types (*β*_2_) appeared to be increased at highly disturbed sites (Dano area). Hence, overall gamma diversity (γ) did not differ significantly among areas, but was slightly higher for the low disturbed savannas (Nazinga area). The referring NMDS ordinations revealed a successive separation of bee communities found in medium (Bontioli area) and highly (Dano area) disturbed savannas from bee communities found in their adjacent cotton and sesame fields (Figure 4b–d).

**Figure 5:**
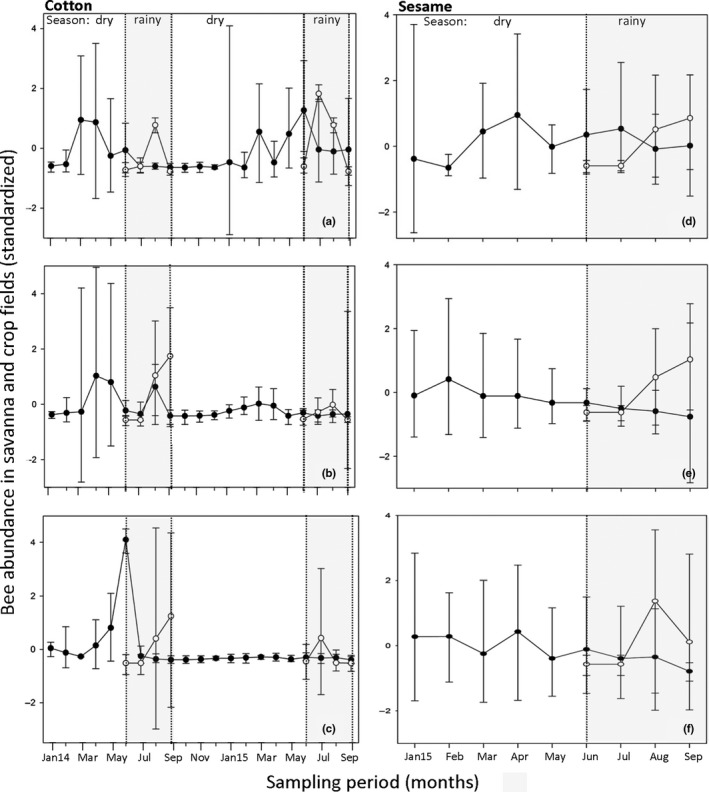
Multiple line and scatter plot with error bars (median and 95% confidence interval) of bee abundance per month (data were standardized prior to analysis) at 12 savanna sites of 1 ha each (filled circles) and 11 cotton and 11 sesame fields (empty circles) in the Southwest of Burkina Faso. Only abundances of bee species occurring at both savanna sites and crop fields in each region were considered (low disturbance—Nazinga: Figure 5a,d; medium disturbance—Bontioli: Figure 5b,e; high disturbance—Dano: Figure 5c,f). Bee data from the cotton fields could only be collected during the rainy seasons of both years from June to September during the flowering period of the crop, as fields lay fallow during the rest of the year. Bee data in sesame fields were collected in 2015 only; hence, the spillover is only plotted for the sampling period in 2015. The sampling period for the savanna‐cotton spillover started in January 2014 (Jan14), for the savanna‐sesame spillover in January 2015 (Jan15)

Savanna total bee abundance peaked during dry seasons in 2014 and 2015. During the rainy season however, bee abundance in cotton and sesame fields was higher than in the savanna habitats regardless of DI (see Figure 5). A movement of bee species from savanna into crop fields (across‐habitat spillover) was suggested by comparing bee abundances in both sampling years 2014 and 2015 in cotton (Figure 5a–c), and in 2015 also in sesame (Figure 5d–f) for all three study areas. The abundance values refer to bee species that occurred in both savanna and crop fields. In the low disturbed sites (Nazinga area) 34 bee species moved between savanna and cotton fields, 24 bee species moved between savanna and sesame fields. We found spillover for a similar number of bee species at medium disturbed sites (Bontioli area, cotton: 35 and sesame: 17 bee species). At highly disturbed sites (Dano area) less bee species were moving between savannas and crop fields (cotton: 25 and sesame: 13 bee species). In cotton, most bee species moving between habitats belong to the family Apidae, followed by Halictidae. This included the most abundant species *A. mellifera*,* H. gribodoi* and *T. fraterna*. Bees moving to cotton fields are mostly generalist, polylectic social bees. Only the long‐horned generalist bee species *T. fraterna* is a solitary bee. In sesame, bees from the family Megachilidae were as abundant as those belonging to the family Apidae. The majority of bees moving to sesame fields are large solitary bees (see Supporting Information Table S3 for bee species identity and abundance in savanna and crop fields).

## 4 DISCUSSION

Our study revealed that bee abundance was highest at intermediate disturbance in the rainy season. Species richness and evenness did not differ significantly at all disturbance intensities. Bee communities at medium and highly disturbed savanna sites comprised only subsets of those at low disturbed sites. Across‐habitat spillover of bees (mostly abundant social bee species) from savanna into crop fields was observed during the rainy season when crops are mass‐flowering whereas most savanna plants are not in bloom.

We assessed bee species communities of savanna habitats with varying disturbance intensities. Among a total of 97 bee species recorded at all our study sites two species were dominant: the stingless bee species *H. gribodoi* and the western honeybee *A. mellifera*. The finding is not surprising as both species are widely distributed across the tropics and known to be eusocial bee species that live in large colonies (Gupta, 2014). Most individuals of both species were caught within the medium disturbed Bontioli area that was characterized by intense agricultural land use including intense apicultural activities. *Hypotrigona gribodoi* is a generalist in terms of food and nesting resources. It seems to benefit from human‐disturbed areas and even nests in walls and under the roofs of huts. No effects of varying DI could be observed in *A. mellifera*. This is likely due to its broad diet, longer foraging ranges compared to most solitary bees, and its ability to locate and utilize discrete patches of resources in the wider landscape, as it efficiently uses scouting (Steffan‐Dewenter & Kuhn, 2003; Steffan‐Dewenter, Muenzenberg, Buerger, Thies, & Tscharntke, 2002). Bee abundance was highest at medium disturbed savannas (Bontioli area). This effect persisted even after excluding the most abundant bee species (*A. mellifera* and *H. gribodoi*) from overall bee abundance data. The observation could be explained by a combination of bee‐friendly habitats found at the study site. The Bontioli area was embedded in agriculture‐bound landscapes with a heterogeneous small‐scale matrix of fields, savanna fragments and home gardens that offered abundant and diverse floral resources to bees (Winfree, Griswold, & Kremen, 2007). In contrast, low (Nazinga area) and highly (Dano area) disturbed savannas were characterized by lower bee species abundance. Our result of significantly decreasing bee abundance in savannas during the rainy season, regardless of DI, could be explained by the same reason, as savanna‐surrounding fields offer floral resources from crop species and vegetables such as chili, tomato, eggplant, okra, squash and pumpkins, whereas the majority of savanna woody plants flower during the dry season. In support of this, studies in Kenya revealed that highest abundance of bees was found in forest edge and farmland habitats with higher amounts of flowers and a more homogeneous distribution of food resources in space and time compared to forest sites (Chiawo, Ogol, Kioko, Otiende, & Gikungu, 2017; Gikungu, Wittmann, Irungu, & Kraemer, 2011; Hagen & Kraemer, 2010). In the highly disturbed savannas (Dano area) lower bee abundance might have been caused not only by limited floral resources, but also by habitat destruction. Therefore, habitat heterogeneity including seminatural savannas and agricultural areas could be the strongest driver for bee abundance in medium disturbed areas. However, another explanatory approach might me that generalist bee abundances where artificially increased through crop‐flower availability over the last years and hence it seems that there are scarce resources in savannas while in reality there are artificially high numbers of bees.

Furthermore, the analysis, if bee species' abundance, richness and evenness in agricultural fields can be related to bee communities in the neighboring savanna habitats (i.e., via across‐habitat movement of bee species), revealed that crop type matters. The significantly positive correlation between bee species' abundance, richness and evenness in savannas and cotton fields but not in sesame fields indicates that both crop types differed regarding their attractiveness to bees or that the surrounding landscape affected bee communities of cotton and sesame fields not consistently (Williams & Winfree, 2013). In a study of landscape effects on bees in Mango (*Mangifera indica* L.) orchards in central Thailand, the authors reported that characteristics of the overall bee community were associated more with farm scale factors than with landscape factors (Tangtorwongsakul, Warrit, & Gale, 2018). Hence, our results show that depending on crop type, bee communities of savanna habitats can impact bee community structure in agricultural fields and vice versa (Klein, Steffan‐Dewenter, & Tscharntke, 2003b; Ricketts, 2004).

The spatial heterogeneity of bee communities found in savannas or crop fields (*β*_1_) in the low disturbed area of Nazinga was higher than that of bee communities found in the medium (Bontioli area) and highly disturbed savannas (Dano area). And furthermore, bee communities of savannas and crop fields embedded within more disturbed landscapes showed a decrease of compositional similarity and consequently an increased species turnover from one habitat to another (*β*_2_). This result emphasizes the importance of conserving natural savanna habitats within agricultural landscapes to maintain a diverse bee species pool that can be crucial for adjacent agricultural production sites (Chiawo et al., 2017). Winfree et al. (2009) carried out a meta‐analysis of 54 published studies recording bee abundance and species richness as a function of human disturbance, clearly revealing that anthropogenic disturbance, in particular habitat destruction, had a significant reducing effect on unmanaged bee species richness. At the medium (Bontioli area) and highly disturbed (Dano area) sites, most of the savanna habitats have been converted into farmland or are being intensively used for timber extraction and grazing, leaving only small fragments of near‐natural savanna. Hence, the decrease in spatial species heterogeneity within one habitat type might be due to habitat simplification whereas the increase in spatial species heterogeneity from one habitat to another appears to be a result of landscape fragmentation that affects ecological connectivity and species exchange among habitats. The low number of savanna sites and fields per DI class might account for nonsignificant values of diversity.

Finally, we analyzed seasonal movements of bees from savanna into crop fields. Our results support the assumption that a seasonal across‐habitat spillover of bees occurred during the rainy season when crops were mass‐flowering. Both agricultural systems, namely cotton and sesame fields, can be regarded as important food resources for bees in times when food resources in the savanna are scarce. In fact, the majority of the melittophilous savanna plants in Burkina Faso are in flower during the dry season or at the very beginning of the rainy season (Arbonnier, 2000). Blitzer et al. (2012) highlighted that spillover from natural to managed habitats is more likely to occur in small‐scale heterogeneous agricultural areas with integrated crop and noncrop ecosystems as it is to be found in Burkina Faso. Agricultural areas cannot only serve as important food resources for bees but at the same time economically benefit from pollination. A study on bee pollinators of cotton and sesame in the same study region revealed that *T. fraterna* and *A. mellifera* were the most efficient pollinators in terms of fruit set and quality of cotton and sesame. Pollination by bees can significantly increase yield quantity and quality with up to 62%, while exclusion of pollinators can cause a yield gap of around 37% in cotton and 59% in sesame (Stein et al., 2017).

## 5 CONCLUDING REMARKS

In our studied agroecological systems, bee species responded differently to varying land‐use intensity with mostly social bees becoming more abundant with increasing habitat disturbance. Despite disturbance intensification, our findings suggest that wild bee communities can persist in anthropogenic landscapes (Basu et al., 2016; Hagen & Kraemer, 2010) and that some species even benefitted disproportionally. West African areas of crop production such as for cotton and sesame may serve as important food resources for bee species in times when resources in the savanna are scarce and receive at the same time considerable pollination service. Our results on spatial and temporal species spillover effects emphasize the importance of natural savanna habitats for conservation and restoration of diverse pollinator communities, which maintain this important ecosystem service. This is of utmost importance in terms of food and income security in sub‐Saharan Africa in general and in Burkina Faso in particular where more than 80 percent of its population relies on agricultural production for their living.

## CONFLICT OF INTEREST

None declared.

## AUTHORS' CONTRIBUTIONS

K. Stein, KEL, and SK designed the study. K. Stein and DC collected data in the field. DC and AP determined bee species and their traits. KD collected and processed land cover data. K. Stein and K. Stenchly analyzed and plotted output data. SP, ISD, and DG contributed toward data analyses and focus of the manuscript. K. Stein wrote the first draft of the manuscript, and all authors contributed substantially to revisions.
