# Supplementary material for: Impact of human disturbance on bee pollinator communities in savanna and agricultural sites in Burkina Faso, West Africa
Source: Ecol Evol. 2018 Jun 17;8(13):6827–38. doi: 10.1002/ece3.4197 (PMC6053565; doi:10.1002/ece3.4197)
Supplement: Supplementary file 1 [file ECE3-8-6827-s001.docx]

**SUPPORTING INFORMATION**

**Table S1.** Test for spatial autocorrelation using Moran's *I* correlation coefficient (observed) with standard deviation (SD) and statistical hypothesis testing considering the expected value of Moran's *I* under the null hypothesis with no spatial autocorrelation (significance threshold *p* ≤ 0.05).

**Table S2.** Total abundance of bee species in savannas of low (Nazinga area), medium (Bontioli area) and high (Dano area) disturbance intensity in the south of Burkina Faso. Data were collected with pan traps for a period of 21 months in 2014 and 2015 covering dry and rainy seasons of both years. Bees were sampled at 12 savanna sites and 11 nearby cotton fields and 11 sesame fields of ca. 1ha each. Bee sampling in the crop fields was carried out only during the rainy seasons from June to September (cotton: 2014 and 2015; sesame: 2015) when crops were in bloom. **(see separate file attached)**

**Table S3.** The 10 most abundant bee species in fields of a) conventional upland cotton (*Gossypium hirsutum*) and b) sesame (*Sesamum indicum*) and their abundance at savanna sites in all three study areas. Data were collected with pan traps for a period of 21 months in 2014 and 2015 (bee sampling in the crop fields only during two rainy seasons from June to October when crops were in bloom) in a total of 12 savanna sites, 11 cotton fields, 11 sesame fields of ca. 1 ha each in the south of Burkina Faso.

| **Bee species** | **Abundance in fields** | **Abundance in savannas** |  |
| --- | --- | --- | --- |
| **a) cotton** |  |  |  |
| *Hypotrigona gribodoi* | 4253 | 21322 |  |
| *Apis mellifera* | 364 | 1548 |  |
| *Tetralonia fraterna* | 120 | 211 |  |
| *Hypotrigona squamuligera* | 49 | 118 |  |
| *Braunsapis* sp.2 | 33 | 169 |  |
| *Plebeina armata* | 26 | 132 |  |
| *Braunsapis* sp.3 | 16 | 276 |  |
| *Liotrigona* sp.2 | 12 | 0 |  |
| *Liotrigona* sp.1 | 9 | 172 |  |
| *Seladonia jucunda* | 8 | 372 |  |
| **b) sesame** |  |  |  |
| *Seladonia lucidipennis* | 294 | 1356 |  |
| *Hypotrigona gribodoi* | 256 | 21322 |  |
| *Apis mellifera* | 142 | 1548 |  |
| *Tetralonia fraterna* | 64 | 211 |  |
| *Plebeina armata* | 32 | 132 |  |
| *Xylocopa scioensis* | 31 | 59 |  |
| *Pseudapis interstitinervis* | 27 | 633 |  |
| *Braunsapis* sp.1 | 26 | 9 |  |
| *Leuconomia bouyssoui* | 26 | 7 |  |
| *Seladonia jucunda* | 26 | 372 |  |
